# Comparison of 3 Dual Single-Pill Combinations for Blood Pressure Lowering: A Secondary Analysis of an International Randomized Trial

**DOI:** 10.1161/CIRCULATIONAHA.126.080934

**Published:** 2026-09-22

**Authors:** Aletta E. Schutte, Abdul Salam, William C. Cushman, H. Asita de Silva, Gian Luca Di Tanna, Diederick E. Grobbee, Krzysztof Narkiewicz, Dike B. Ojji, Neil R. Poulter, Markus P. Schlaich, Wilko Spiering, Bryan Williams, Jackson T. Wright, Thirunavukarasu Kumanan, Peter Hay, Gamini Galappatthy, Tanya Pereira, Faslur Rahuman, Arjuna P. de Silva, Godwin Constantine, Chris Gianacas, Stephen Vander Hoorn, Mathangi Shanthakumar, Xiaoqiu Liu, Nelson Wang, Sonali R. Gnanenthiran, Paul K. Whelton, Anthony Rodgers

**Affiliations:** 1 The George Institute for Global Health, University of New South Wales 7800 , Australia (A.E.S., A.S., C.G., S.V.H., M.S., X.L., N.W., S.R.G., A.R.).; 2 India and Prasanna School of Public Health, Manipal Academy of Higher Education, India (A.S.).; 3 University of Tennessee Health Science Center, Memphis, TN (W.C.C.).; 4 Clinical Trials Unit, University of Kelaniya, Sri Lanka (H.A.d.).; 5 University of Applied Sciences and Arts of Southern Switzerland, Switzerland (G.L.DT.).; 6 Julius Global Health, the Julius Center for Health Sciences and Primary Care, University Medical Center Utrecht, Utrecht University, The Netherlands (D.E.G.).; 7 Medical University of Gdańsk, Poland (K.N.).; 8 Department of Internal Medicine, University of Abuja 99399 , Nigeria (D.B.O.).; 9 Imperial College London, London, UK (N.R.P.).; 10 Dobney Hypertension Centre, Royal Perth Hospital and RPH Research Foundation, The University of Western Australia, Perth, Australia (M.P.S.).; 11 Departments of Cardiology and Nephrology, Royal Perth Hospital, Perth, Australia (M.P.S.).; 12 University Medical Center Utrecht, Utrecht University, The Netherlands (W.S.).; 13 University College London, London, UK (B.W.).; 14 University Hospitals Cleveland Medical Center, Case Western Reserve University, Cleveland, OH (J.T.W.).; 15 Department of Medicine, University of Jaffna, Sri Lanka (T.K.).; 16 Castle Hill Medical Centre, Sydney, Australia (P.H.).; 17 Institute of Cardiology, National Hospital, Colombo, Sri Lanka (G.G.).; 18 Colombo South Teaching Hospital 92954 , Kalubowila, Sri Lanka (T.P., F.R.).; 19 Department of Medicine, University of Kelaniya, Sri Lanka (A.dS.).; 20 Department of Medicine, University of Colombo, Sri Lanka (G.C.).; 21 Department of Epidemiology, Tulane University Celia Scott Weatherhead School of Public Health and Tropical Medicine, New Orleans, LA (P.K.W.).

**Keywords:** antihypertensive agents, blood pressure, drug combinations, hypertension

Recent clinical practice guidelines recommend dual low-dose single-pill combination therapy for initial treatment of hypertension, given the improvements in efficacy, adherence, and reduction in therapeutic inertia that they impart without compromising tolerability.^1^ However, randomized comparisons between different dual single-pill combinations are limited. We therefore report the comparative efficacy of 3 dual combinations, each containing 2 of the major antihypertensive drug classes: an angiotensin receptor blocker (telmisartan), a dihydropyridine calcium channel blocker (amlodipine), and a thiazide-like diuretic (indapamide).

This analysis was embedded in a previously reported trial in 7 countries.^2^ Ethics approval was obtained in each country; all participants provided informed consent. After a 4-week single-blind run-in with low-dose triple therapy (20 mg of telmisartan, 2.5 mg of amlodipine, and 1.25 mg of indapamide), participants were randomized to triple therapy or one of its 3 dual combinations at the same doses as the triple therapy: telmisartan-indapamide (TI), telmisartan-amlodipine (TA), or amlodipine-indapamide (AI). After 6 weeks, doses were doubled unless contraindicated. The primary efficacy outcome was change in mean home systolic blood pressure (BP) from randomization to week 12, analyzed using hierarchical linear modeling with final systolic BP (SBP) as the model outcome and controlling for baseline SBP, treatment arm, and visit. Huber-White sandwich estimation and covariance matrices accounted for clustering within patient and site. The primary safety outcome was treatment withdrawal attributable to adverse events. Because the parent trial was designed to compare triple therapy with each dual therapy, dual-versus-dual comparisons should be interpreted as exploratory (Trial registration: NCT04518293).

Documentation around the analyses presented here will be made available to qualified scientific and medical researchers, upon researcher’s request, as necessary for conducting legitimate research. Patient data will be deidentified and will be shared via a secure means. Requests for data will only be reviewed after approval of the product in the United States and the European Union, and after approval by George Medicines of its data access request and receipt of its executed data sharing agreement. A request form can be obtained by email to the corresponding author or to info@george-medicines.com.

After the 4-week run, 834 participants with an SBP 110 to 154 mm Hg were randomized to dual therapy, with similar baseline characteristics across groups. Mean age was 59 years, 52% were women, and mean clinic BP at screening was 142/85 mm Hg while participants were receiving a mean of 1.6 antihypertensive medications before randomization. Mean home BP at randomization was 129/78 mm Hg after run-in. Adherence to study treatment was 97% (TI, 97%; TA, 97%; AI, 96%), and 95% of participants completed follow-up.

At 12 weeks, TI resulted in greater reductions in home BP compared with TA and AI (Figure). Mean home SBP at week 12 was 127 mm Hg (95% CI, 126–128) in the TI group, 130 mm Hg (95% CI, 129–131) in the TA group, and 129 mm Hg (95% CI, 128–130) in the AI group. The least-squares mean difference in home SBP change from randomization to week 12 was −3.1 mm Hg (95% CI, −4.4 to −1.8; *P*<0.001) for TI versus TA and −2.1 mm Hg (95% CI, −3.4 to −0.7; *P*=0.003) for TI versus AI. Similar differences were observed for home diastolic BP. These effects were consistent across dose levels and evident at week 6.

**Figure.**
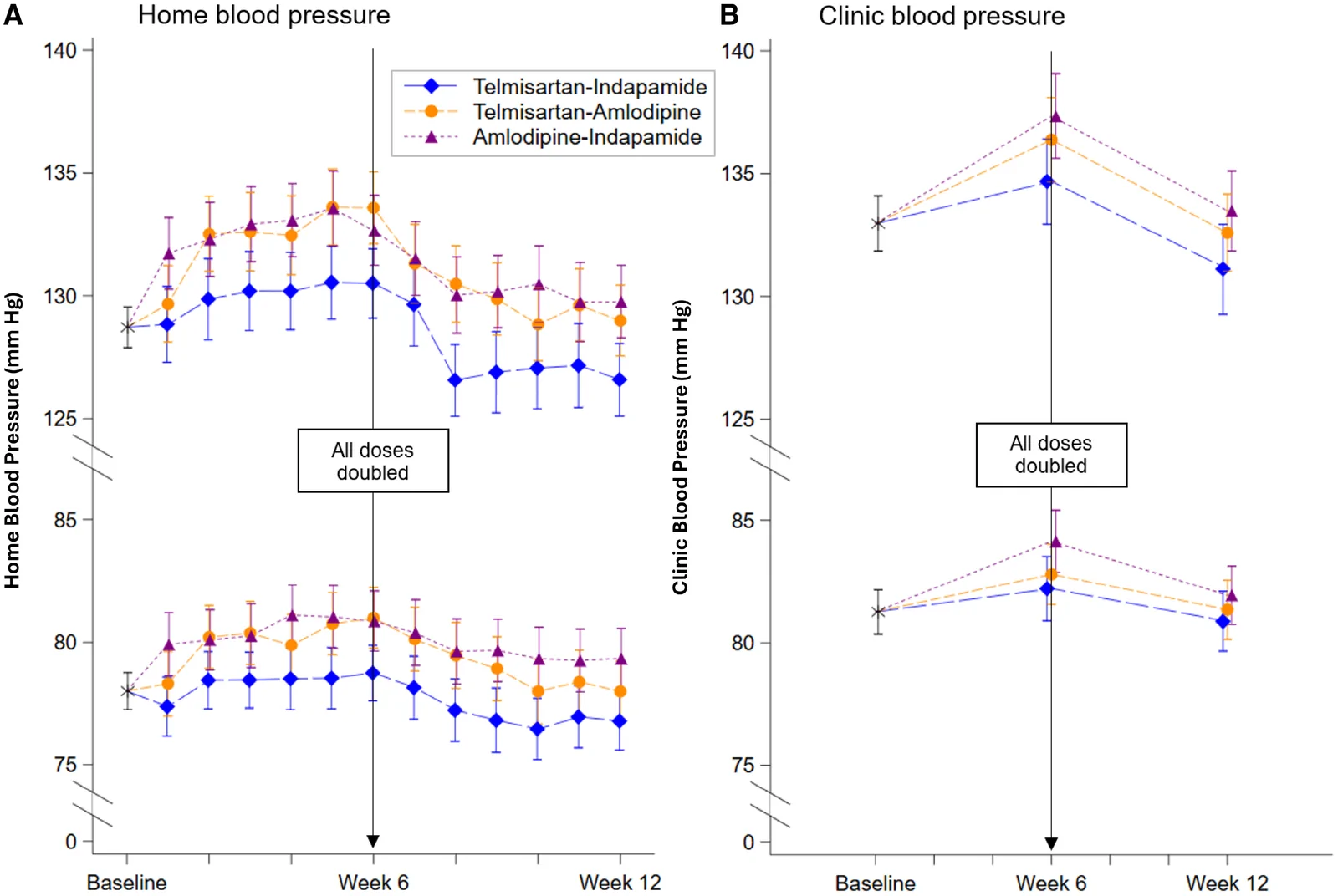
**Home (A) and clinic mean blood pressure (B; 95% CI) over time, by group.** All participants received a 4-week run-in with low-dose triple therapy before randomization; baseline values shown reflect measurements obtained at the end of the run-in period.

Home BP control (<130/80 mm Hg) at week 12 was achieved in 44% in the TI group compared with 39% in the TA group and 33% in the AI group. Clinic BP control (<130/80 mm Hg) at week 12 was 28%, 23%, and 21% in the TI, TA, and AI groups, respectively, with corresponding control (<140/90 mm Hg) at 61%, 61%, and 53%.

All treatments were well tolerated. Treatment withdrawal because of adverse events occurred in 1.4%, 1.1%, and 1.4% of participants in the TI, TA, and AI groups, respectively. Serious adverse events were infrequent and similar across groups. Symptomatic hypotension occurred in 4.0%, 1.8%, and 1.4% of participants in the TI, TA, and AI groups, respectively. Hypokalemia (potassium <3.5 mmol/L) occurred in 4.7%, 0%, and 12.7% of participants in the TI, TA, and AI groups, respectively. There were no meaningful differences between groups in measures of kidney function or orthostatic BP changes.

In this randomized trial involving participants from multiple countries, the dual combination of TI was more effective in lowering home BP than TA or AI. This difference was consistent across home SBP and diastolic BP, as well as home BP control rates, but was less apparent for clinic BP. All treatments were associated with low rates of treatment discontinuation and were well tolerated.

Although statistically significant, the differences in BP reduction between combinations were modest (approximately 2 to 3 per 1.5 mm Hg). Previous randomized comparisons of dual therapies have similarly shown modest differences between combinations at comparable dose levels.^3,4^ Based on previous meta-analyses, differences of this magnitude would be expected to translate into small differences in cardiovascular risk reduction (approximately 4% to 6%), likely modest for individuals but potentially relevant at population level. More broadly, overall BP reduction is primarily determined by the number of drug classes,^5^ and triple therapy has ≈5 mmHg superior BP reduction to dual therapy.^5^

Strengths of our analysis include the randomized, double-blind design, inclusion from multiple countries, and use of home BP measurements, which provide more precise estimates of BP changes. Limitations include that this was a secondary analysis of a trial not specifically powered for comparisons between dual combinations, that antihypertensive dose levels were limited, that analyses were not prespecified with potential type I error inflation attributable to multiple comparisons, and that the run-in phase may have selected participants more tolerant of combination therapy, limiting generalizability and underestimating adverse events. Clinic BP comparisons were less precise than home BP measurements. Findings by region and ethnicity performed in the parent trial were broadly consistent.^2^

In conclusion, TI provided slightly greater BP reduction than TA or AI at the doses tested in this population. However, these exploratory comparisons were not prespecified or powered. The modest differences observed suggest that all major dual combinations remain clinically appropriate.

## Article Information

### Acknowledgments

The authors thank the staff who contributed to the conduct of the trial, the Data Safety and Management Board, and the patients who volunteered to participate in the study. The authors also thank Suzanne Oparil, PhD, for her key contributions to this work.

### Author Contributions

A.E.S. wrote the first draft with input from A.S., P.K.W., N.W., and A.R. Statistical analyses were conducted by G.L.D.T., M.S., C.G., S.V.D.H., X.J., and the Veristat team acknowledged below. The steering committee had full access to all data. All authors commented and edited on the draft manuscript and supported the decision to submit.

### Disclosures

Several authors are employed at The George Institute for Global Health (TGI), which holds an interest in George Medicines Pty Ltd (GM) via its social enterprise arm, George Institute Ventures. None of the TGI staff have a personal financial interest in GM. Dr Rodgers is seconded part-time to GM. TGI holds patents for ultra-low-dose fixed-dose combination products for the treatment of hypertension and diabetes, and Dr Rodgers is listed as one of the inventors (granted: US 10,369,15; US 10,799,487; US 10,322,117; US 11,033,544; and US 11,478,462; Pending: US 17/932,982; US 18/446,268; US 17/598,122; US 17/317,614; US 17/527,084; US 17/527,085; and US 17/527,087). Dr Rodgers does not have a financial interest in these patents. None of the other authors have a financial interest in GM or have received funding for their independent contribution to the GMRx2 program. Dr Schutte has received consulting fees or speaker honoraria from Omron Healthcare, Medtronic, Servier, Novo Nordisk, Novartis, Roche, Alnylam, Biozen, AstraZeneca, and Sky Labs. She holds the following roles: co-chair, National Hypertension Taskforce Australia; board member, Hypertension Australia; and board member, Australian Cardiovascular Alliance. She is supported by an investigator grant from the National Health and Medical Research Council of Australia (GNT 2017504). Dr Rodgers has received consulting fees as a data and safety monitoring board member for Idorsia. He was supported by a fellowship grant from the National Health and Medical Research Council of Australia (GNT 1160743). Dr Ojji has received speaker honoraria from Novartis, AstraZeneca, Servier, Swipha, and Boehringer for speaking at educational meetings. Dr Narkiewicz has received speaker and consulting honoraria from Bausch Health, Berlin-Chemie/Menarini, Egis, Idorsia, Janssen, Gedeon Richter, Gilead, Krka, Novo Nordisk, Polpharma, Recordati, Sandoz, and Servier. He is on advisory boards for Polpharma and Zentiva and has received honoraria. Dr Schlaich has received consulting fees or travel and research support from Medtronic, Abbott, ReCor, Novartis, Servier, Pfizer, and Boehringer-Ingelheim. He is the current president of Hypertension Australia and treasurer of the World Hypertension League. Dr Cushman has received institutional grants from George Medicines and consulting fees from Alnylam Pharmaceuticals, Idorsia, and Azurity Pharmaceuticals. Dr Poulter has received financial support from several pharmaceutical companies that manufacture blood pressure–lowering agents for consultancy fees (Servier and Aktiia), research projects and staff (Servier and Pfizer), and for arranging and speaking at educational meetings (AstraZeneca, Lri Therapharma, Napi, Servier, Sanofi, Eva Pharma, Pfizer, Emcure India, Dr Reddy’s Laboratories, and Zydus). He was supported by the National Institute for Health Research senior investigator awards, Biomedical Research Centre funding, and the British Heart Foundation Research Centre excellence award. Dr Grobbee currently receives funding to his department as part of the European Union Horizon Hypermarker project. He has no other disclosures relevant to this report. Dr Wright has received institutional grants from the National Institutes of Health, Ohio Department of Medicaid, and the Agency for Healthcare Research and Quality. He has attended an advisory board meeting for Medtronic, Inc and received travel support. He is a chair on the data and safety monitoring board for IMPACTS MIND trial (Implementation of Multifaceted Patient-Centered Treatment Strategies for Intensive Blood Pressure Control to Minimize Cognitive Decline) at Tulane University, New Orleans, LA. He is on the Northeast Ohio Neighbourhood Health Center community board of directors. Dr Spiering currently receives funding to his department as part of the European Union Horizon Hypermarker project. He is on the advisory board of Neuroloop.

## References

[1] Lauder L Weber T Böhm M Brouwers S Bruno RM Gerdts E Kreutz R Lüscher TF Mancia G McEvoy JW. European guidelines for hypertension in 2024: a comparison of key recommendations for clinical practice. Nat Rev Cardiol. 2025;22:675–688. doi: 10.1038/s41569-025-01187-2 10.1038/s41569-025-01187-2 40691360

[2] Rodgers A Salam A Schutte AE Cushman WC de Silva HA Di Tanna GL Grobbee DE Narkiewicz K Ojji DB Poulter NR; GMRx2 Investigators. Efficacy and safety of a novel low-dose triple single-pill combination of telmisartan, amlodipine and indapamide, compared with dual combinations for treatment of hypertension: a randomised, double-blind, active-controlled, international clinical trial. Lancet. 2024;404:1536–1546. doi: 10.1016/S0140-6736(24)01744-6 10.1016/S0140-6736(24)01744-6 39426836

[3] Ojji DB Mayosi B Francis V Badri M Cornelius V Smythe W Kramer N Barasa F Damasceno A Dzudie A; CREOLE Study Investigators. Comparison of dual therapies for lowering blood pressure in Black Africans. N Engl J Med. 2019;380:2429–2439. doi: 10.1056/NEJMoa1901113 10.1056/NEJMoa1901113 30883050

[4] Prabhakaran D Roy A Chandrasekaran AM Kondal D Mukherjee S Kiru G Singh K Salwa H Sobitharaj EC Lobo AS; TOPSPIN Clinical Consortia. Comparison of dual therapies for hypertension treatment in India: a randomized clinical trial. Nat Med. 2025;31:3169–3175. doi: 10.1038/s41591-025-03854-w 10.1038/s41591-025-03854-w PMC12443627 40715816

[5] Wang N Salam A Pant R Kumar A Dhurjati R Haghdoost F Vidyasagar K Kaistha P Esam H Gnanenthiran SR. Blood pressure-lowering efficacy of antihypertensive drugs and their combinations: a systematic review and meta-analysis of randomised, double-blind, placebo-controlled trials. Lancet. 2025;406:915–925. doi: 10.1016/S0140-6736(25)00991-2 10.1016/S0140-6736(25)00991-2 40885583

